# Obesity associated with increased brain age from midlife

**DOI:** 10.1016/j.neurobiolaging.2016.07.010

**Published:** 2016-11

**Authors:** Lisa Ronan, Aaron F. Alexander-Bloch, Konrad Wagstyl, Sadaf Farooqi, Carol Brayne, Lorraine K. Tyler, Cam-CAN, Paul C. Fletcher

**Affiliations:** a Brain Mapping Unit, Department of Psychiatry, University of Cambridge, UK; b Yale School of Medicine, Yale University, USA; c Department of Clinical Biochemistry, Institute of Metabolic Sciences, Cambridge, UK; d Institute of Public Health, University of Cambridge, Cambridge, UK; e MRC Cognition and Brain Sciences Unit, Cambridge Center for Ageing and Neuroscience (Cam-CAN), Cambridge, UK

**Keywords:** Obesity, White-matter volume, Structural MRI, Population-based

## Abstract

Common mechanisms in aging and obesity are hypothesized to increase susceptibility to neurodegeneration, however, direct evidence in support of this hypothesis is lacking. We therefore performed a cross-sectional analysis of magnetic resonance image-based brain structure on a population-based cohort of healthy adults. Study participants were originally part of the Cambridge Centre for Ageing and Neuroscience (Cam-CAN) and included 527 individuals aged 20–87 years. Cortical reconstruction techniques were used to generate measures of whole-brain cerebral white-matter volume, cortical thickness, and surface area. Results indicated that cerebral white-matter volume in overweight and obese individuals was associated with a greater degree of atrophy, with maximal effects in middle-age corresponding to an estimated increase of brain age of 10 years. There were no similar body mass index-related changes in cortical parameters. This study suggests that at a population level, obesity may increase the risk of neurodegeneration.

## 1 Introduction

The link between obesity and adverse health outcomes such as diabetes, cancer, and cardiovascular disease is well established and poses a major challenge to current and future health care provision. Moreover, it is increasingly recognized that obesity may act to accelerate or advance the onset of age-related changes such as neurodegeneration, either directly or through associated comorbidities (Doherty, 2011). These associations, taken together with the increased rate of obesity in elderly populations (Flegal et al., 2012) render it critical to understand the full impact of obesity on brain health, in particular as evidence suggests that adverse outcomes may be mitigated through intervention (Gunstad et al., 2011).

A number of strands of evidence have related biological processes associated with obesity to changes found in normal aging. For example, as with normal aging, obesity increases oxidative stress (Furukawa et al., 2004) and promotes inflammation through the production of proinflammatory cytokines produced in adipose tissue (Arnoldussen et al., 2014, Chung et al., 2009). In turn, cytokines and proinflammatory markers such as interleukin 6 and tumor necrosis factor-α have been linked to cognitive decline (Chung et al., 2009, Griffin, 2006, Wilson et al., 2002) and have been shown to be upregulated in regions undergoing neurodegeneration (Wilson et al., 2002). Inflammatory biomarkers have been associated with increased brain atrophy, a common marker of aging (Jefferson et al., 2007), as have other endophenotypes such as shortened telomere length (Wikgren et al., 2014). Conversely, a considerable body of evidence exists suggesting that caloric restriction may be neuroprotective, leading to a delay or slowing of aging (Colman et al., 2014, Colman et al., 2009, Masoro, 2005, Sohal and Weindruch, 1996), a reduction in age-related apoptosis (Someya et al., 2007), and age-related production of proinflammatory cytokines (Kalani et al., 2006, Spaulding et al., 1997).

In short, the growing body of literature that relates common markers of aging to those observed in obesity supports the hypothesis that obesity may accelerate or advance the onset of brain aging. However, direct studies in support of this link are lacking. For example, although many studies have reported a link between increased body mass index (BMI) and declining cognitive function (Cournot et al., 2006, Debette et al., 2011), as well as increased risk of dementia and Alzheimer's Disease (Gustafson et al., 2004, Whitmer et al., 2005, Xu et al., 2011), other studies contradict these findings (Qizilbash et al., 2015), and indeed, it has even been suggested that lower, rather than higher, body mass may be predictive of the onset of AD in the years immediately preceding the onset of clinical symptoms (Fielding et al., 2013, Knopman et al., 2007). The literature on brain structural changes too is complex. Although many studies report a negative correlation between BMI and gray matter volume (GMV) (increased BMI linked to lower GMV) (Brooks et al., 2013, Debette et al., 2014, Gunstad et al., 2008, Hassenstab et al., 2012, Veit et al., 2014), other reports are contradictory (Haltia et al., 2007, Pannacciulli et al., 2007, Sharkey et al., 2015). More significantly, despite a considerable number of often highly powered studies across the adult lifespan (Taki et al., 2008), there is a conspicuous lack of either global findings related to obesity or evidence of an aging interaction (for a review, see Willette and Kapogiannis, 2015).

Thus, although current neuroimaging evidence certainly suggests altered brain structure is associated with obesity, it fails to support the hypothesis that obesity influences age-related atrophy of the brain. There are a number for reasons for why this might be. Different tissue types in the brain age at different rates (Walhovd et al., 2005), perhaps limiting the sensitivity of cross-sectional studies over limited age-periods. Moreover, there is a complex and somewhat compensatory interaction between the change in cortical thickness and surface area (Storsve et al., 2014), that may confound analysis by morphometric methods such as voxel-based morphometry commonly employed in structural studies of obesity. In addition, voxel-based morphometry methods are designed to obviate global changes in favor of regional analyses. If obesity, like aging affects the brain globally, it may be the case that a significant global interaction may be obfuscated. Analysis of white matter too may be confounded. Although some studies suggest obesity and inflammation are both associated with smaller fractional anisotropy in diffusion tensor imaging (Stanek et al., 2011, Verstynen et al., 2013), it is also the case that additional factors related to obesity and aging such as blood pressure are positively associated with fractional anisotropy (Verstynen et al., 2013), raising the possibility that competing effects may hamper identification of an age-by-BMI interaction. The alternative to these propositions is that obesity may increase the rate of aging of brain tissue but that these effects are subtle and within the scope of normal aging parameters.

In this cross-sectional population-based study, we assessed the impact of obesity on brain structure across the adult lifespan using global parameters of volume, cortical thickness, and surface area. The goal of our study was to establish the overall effect of obesity on gray (i.e., cortical thickness and surface area) and white matter; to determine whether obesity affected tissue types differentially; and crucially to investigate whether obesity was associated with an increase in brain age, evaluated with reference to lean controls. We were particularly interested in whether changes associated with obesity (i.e., deviations from lean age-matched controls) might occur during a particular vulnerable period.

## 2 Materials and methods

### 2.1 Subjects

A total of 527 subjects with an age range of 20–87 years were included in this study. Participants were cognitively healthy adults recruited from the local community over a period of 5 years as part of an ongoing project to investigate the effects of aging on memory and cognition at the Cambridge Centre for Aging and Neuroscience (Shafto et al., 2014). Ethical approval for the Cam-CAN study was obtained from the Cambridgeshire 2 (now East of England–Cambridge Central) Research Ethics Committee. Of these, 54 subjects were excluded on the basis of being underweight (BMI < 18.5kgm^−1^), under the age of 20, or for reasons of poor MR image quality (see below). Subject demographics are detailed in Table 1. The mean age was 54 years (range 20–87), and mean BMI was 26 kg/m^2^ (18.5–45.5). The final cohort included 246 (51%) lean controls (BMI between 18.5–25 kgm^−2^), 150 overweight subjects (31%; BMI 25–30 kgm^−2^), and 77 obese subjects (BMI >30 kgm^−2^). There was a significant positive correlation between age and BMI (r = 0.24, *p* < 0.001). Various health and lifestyle factors were recorded including self-reported history of diagnosis of diabetes, stroke, cancer, myocardial infarction, high blood pressure, and high cholesterol. A self-report questionnaire was used to calculated total estimated physical activity per week (measures as kJ/d/Kg). Education level was binarized to those with or without degree-level qualifications. Gross household income was also included, defined as those above and below a threshold income of £30,000. There were no incidences of Parkinson's disease or multiple sclerosis. Cognitive performance was quantified using Cattell Culture Fair (scale 2, form A) (Shafto et al., 2014).

### 2.2 MR acquisition and image analysis

#### 2.2.1 MR acquisition

Structural images were acquired on a 3T Siemens TIM Trio system employing a 32-channel head coil. A high resolution 3D T1-weighted structural image was acquired using a magnetization prepared rapid gradient echo sequence with the following parameters: repetition time = 2250 milliseconds; echo time = 2.99 milliseconds; inversion time = 900 milliseconds; flip angle = 9°; field of view = 256 mm × 240 mm × 192 mm; voxel size = 1 mm isotropic; GRAPPA (generalized autocalibrating partially parallel acquisitions) acceleration factor = 2; acquisition time of 4 minutes and 32 seconds.

#### 2.2.2 Cortical reconstruction and structural measures

**Fig. 1:**
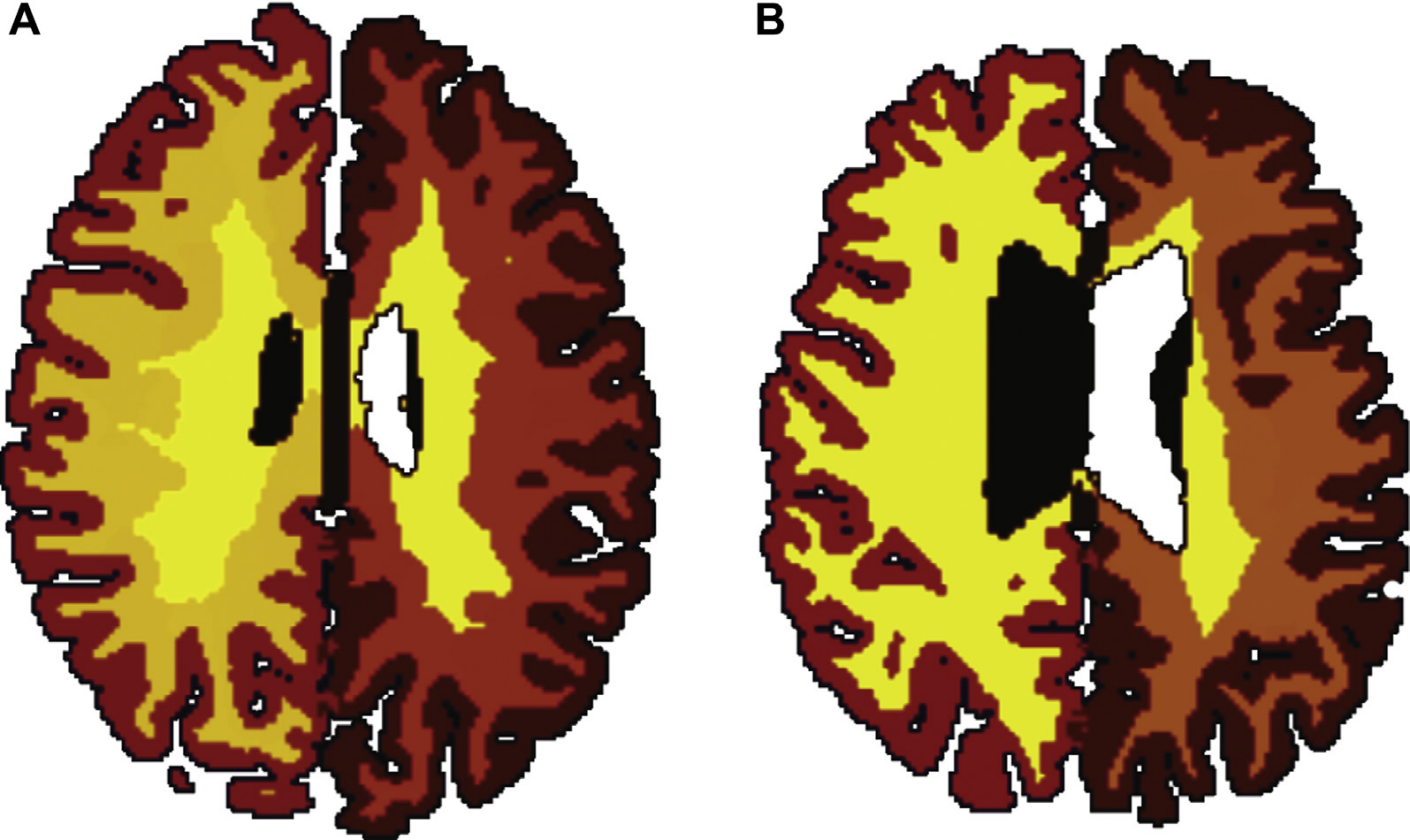
Example of gray and white-matter segmentations in FreeSurfer for, sex-matched subjects (A) lean (56 years, BMI 19.5) and (B) obese (50 years, BMI = 43.4).

Cortical reconstructions were generated using the software FreeSurfer (Dale et al., 1999, Fischl et al., 1999a, Fischl and Dale, 2000). The FreeSurfer program was specifically developed for cortical reconstruction and has been extensively validated (Han et al., 2006, Kuperberg et al., 2003, Rosas et al., 2002). Measures of cerebral white-matter volume and intracranial volume were generated. We further quantified whole-brain cerebral surface area, which was based on the pial surface, and whole-brain cortical thickness, which was taken as the mean thickness across each hemisphere, where thickness was first estimated at each vertex in the reconstruction measured as the minimum distance between the gray–white and pial surfaces. Surface reconstruction processes were conducted in native space. Examples of gray-white-matter segmentation for representative age-matched lean and obese subjects are included in Fig. 1. All reconstructions were quality controlled (see below).

#### 2.2.3 Quality assurance

All reconstructions were qualitatively assessed by a single rater (LR) and categorized as “good” (n = 411, 81%), “adequate” (n = 62, 12%), or “poor” (n = 33, 7%). There was a statistically significant interaction between age and quality of surface reconstruction (z = −8.6, *p* < 0.001), with older subjects more likely to have poor reconstruction quality.

Because manual edits of the entire data set was unfeasible, we decided to test the effect of edits on a subsample of the data. For this, manual edits were done on 100 brains chosen at random, and the cortical reconstructions recomputed. New values of cortical surface area, thickness, and white-matter volume were these generated and contrasted to the original, unedited values. Bland and Altman plots (Supplementary Figure 1) and linear regression were used to assess the variability and bias of values pre-editing and postediting (Bland and Altman, 1986). Results suggest that for reconstructions deemed “good” and “adequate”, editing did not statistically significantly affect morphometric values (white-matter volume F = 2.9, *p* = 0.09; surface area F = 1.7, *p* = 0.2; thickness F = 0.7, *p* = 0.4). The mean difference between pre-edits and postedits for each measure was zero, indicating no bias between measurements. On this basis we excluded all reconstructions deemed “poor” (n = 33).

#### 2.2.4 Regional analysis of thickness and surface area

Cortical thickness was further explored at a regional level using FreeSurfer. Each individual cortical reconstruction was aligned to a template using a hierarchical spherical averaging method (Fischl et al., 1999b). Thereafter, group (lean vs. overweight or obese)-by-age interactions were explored using a general linear model with total intracranial volume and gray-white-matter contrast (see Section 2.3 below) as covariates. Monte Carlo correction (10,000 iterations, *p* < 0.01) was used to account for multiple comparisons at the cluster-level.

### 2.3 Statistical analysis

Previous studies have demonstrated although the cortex ages linearly, white-matter volume has a nonlinear aging trajectory. For this reason, we used penalized spline mixed-effect models to describe the age-dependent variation in each measure. Details of these methods have been described elsewhere (Alexander-Bloch et al., 2014, Wood and Scheipl, 2015). Data were Box-Cox-transformed and mean-centered where appropriate to control for non-normal distribution and mean-centering, respectively. All analysis was done in R (version 3.2, www.cran.r-project.org) using the packages *nlme, methcomp, and gamm4* (Wood and Scheipl, 2015).

All brain parameters were controlled for the effects of sex and total intracranial volume (derived from the FreeSurfer pipeline). There were no hemispheric differences for any measure (i.e., white-matter volume, cortical surface area, and thickness); thus, left and right data of each measure were aggregated into a single value per subject. Independent parameters for the following were included as regressors: self-reported diagnosis of high blood pressure, diabetes, cancer, myocardial infarction, stroke, and high cholesterol. Sociodemographic parameters such as education level as well as household income were additionally included, as were self-reported levels of physical activity per week. The numbers of individuals who described themselves as current smokers were low (Table 1) and did not differ across groups, thus smoking was not included as a covariate. Mindful of the potential confound of cognitive decline in older subjects, we repeated our regression analysis using cognitive scores from the Cattell measure as an additional regressor.

As well as total intracranial volume, cortical thickness was additionally corrected for gray–white-matter percentage. This latter parameter is derived from the gray-scale values of cortical gray matter and the cerebral white-matter and is used as a surrogate of myelination changes, which are hypothesized to affect the contrast between tissue types and thus may confound measures of thickness (Grydeland et al., 2013, Storsve et al., 2014, Westlye et al., 2009).

#### 2.3.1 Estimating brain age in lean and overweight/obese

To compute the white matter–related age difference between lean and overweight or obese, we again divided the data into 2 categories based on weight (i.e., lean vs. overweight or obese). We then used spline methods (see above) to model the white-matter volume for each group. In turn, these models were used to estimate the difference in brain age between the 2 groups. To do this, we calculated the mean difference in age between the groups for every white-matter volume. For example, for the volume 445 cm^3^, the model for lean subjects indicated a corresponding age of 60 years, whereas the model for overweight or obese subjects indicated a corresponding age of 50 years. Thus, we estimated a difference in brain age of 10 years for this age range.

Because of the sensitivity of splines to outliers (Supplementary Figure 2), we further generated CIs for these values. In doing this, analysis was limited to the age range 37–87 years to obviate difficulties in subtracting a maturational increase (in overweight or obese subjects) from a decrease (in lean subjects) (owing to inverted U-shaped trajectory of the data). In other words, we aimed to prevent the situation of comparing data from mature overweight and obese subjects with data from younger, lean adults with the same volume. For example, owing to the inverted-U shape, lean subjects have an average white-matter volume of 445 cm^3^ at 26 years and 60 years, whereas overweight and obese subjects have the same volume at 50 years. Thus, by excluding subjects below 37 years, we can ensure that our calculation of brain age difference between groups is based on subjects with the same degree of maturity. We also set the following limitations (1) prevent bootstrapping from finding ages younger than max of inverted-U; (2) set to zero if obese is larger than normal; and (3) if there is no one old enough, then set to last age when there was someone old enough. Bootstrapping was performed for 10,000 iterations. We then calculate the 95% and 90% CIs.

## 3 Results

### 3.1 White matter volume

**Fig. 2:**
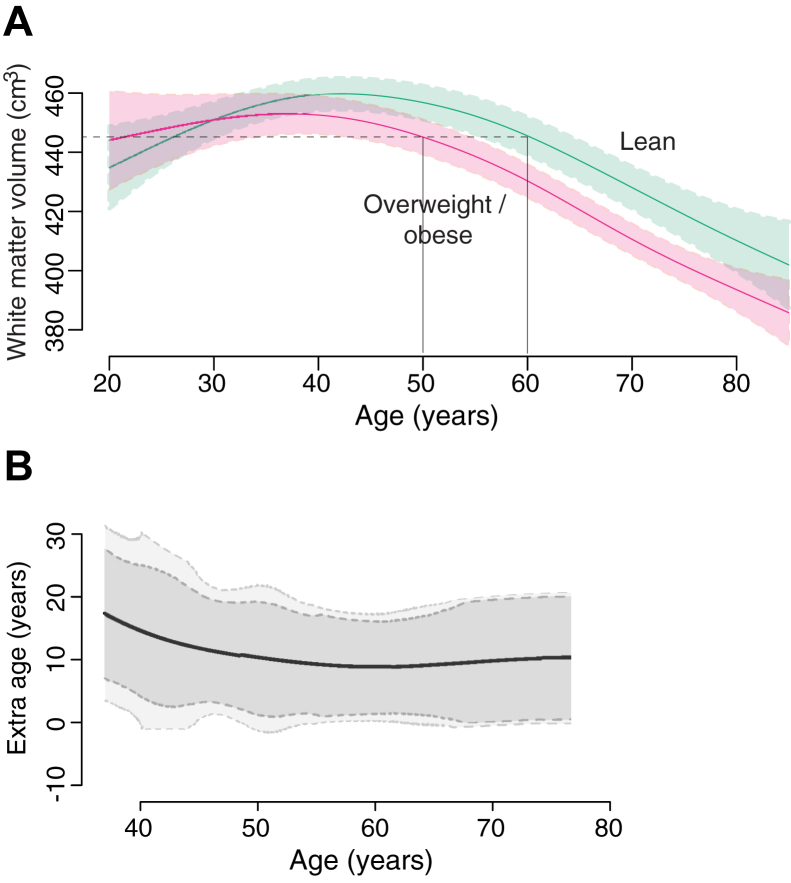
Age-related change in white-matter volume in overweight or obese subjects estimated to equate to an average increase in brain age of 10 years compared to controls. (A) Age-trajectory of white-matter volume for lean (BMI 18.5–25 kgm^−2^), overweight (BMI 25–30 kgm^−2^), and obese (BMI >30 kgm^−2^). The difference in “brain age” between the groups was calculated by comparing the difference in age between groups for each value of white-matter volume. For example, at 50 years, overweight or obese subjects have an estimated volume white-matter volume of 445 cm^3^ whereas lean subjects reach the same volume at the average age of 60 years, equating to an average 10 years increased brain age for overweight or obese subjects. (B) Estimated difference in brain age between lean and overweight or obese subjects based on differences in population models of white-matter volume change with age. 90% and 95% CIs generated using bootstrap methods (10,000 iterations).

In line with previous studies, subjects showed a nonlinear change in white-matter volume with age, increasing to a maximum in middle-age, and decreasing thereafter (Fig. 2A; F = 25, *p* < 0.0001). Critically, there was a statistically significant age:BMI interaction (t = −3, *p* = 0.003). The inclusion of Cattell cognitive scores did not affect this result. Comparing models of white-matter volume between lean and overweight or obese subjects, we estimated an average increase in brain age associated with adiposity of approximately 10 years, with slight increases in middle-age subjects (approximately 40 years) (Fig. 2B).

Detailed examination of the data revealed that a previous diagnosis of elevated cholesterol (as described in self-reported health questionnaire) independently negatively impacted on white-matter volume over and above the effects of age and BMI (t = −2.3, *p* = 0.02), suggesting that some common metabolic comorbidities associated with obesity may have an additional and distinct role in increasing susceptibility to neurodegeneration. However, there was no evidence of a mitigating effect of exercise, income, or education on the BMI-related impact on brain structure when other factors were taken in to account.

### 3.2 Cortical surface area

**Fig. 3:**
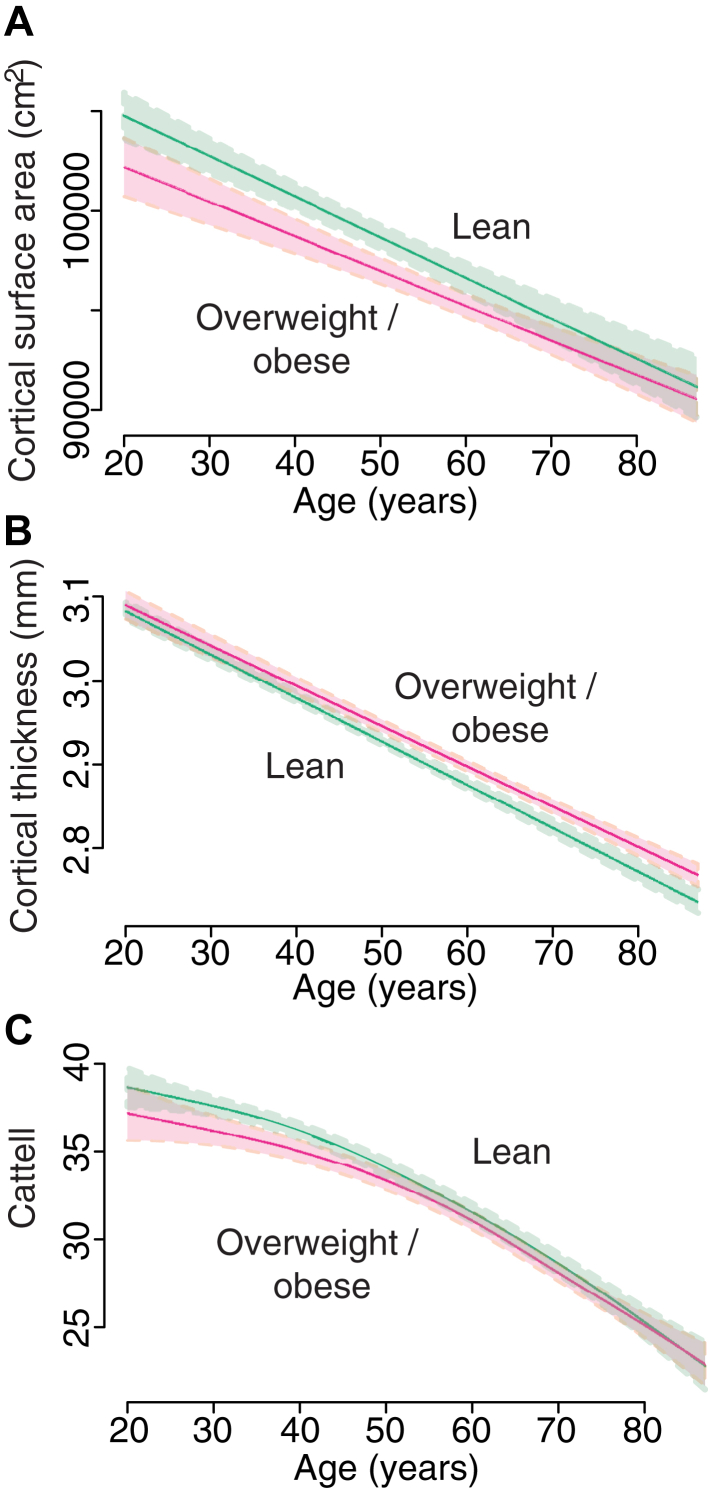
Age-related change in (A) cortical surface area, (B) thickness, and (C) cognitive scores (cattell) contrasted between lean and overweight or obese subjects.

There was a significant negative effect of age on cortical surface area (based on the pial surface) for each adiposity group (F = 191, *p* < 0.0001). However, there was no BMI-related difference in total cortical surface area and no age:BMI interaction (Fig. 3A) even after including Cattell scores as an additional regressor.

### 3.3 Cortical thickness

Like surface area, cortical thickness also decreased in a near-linear trajectory across the lifespan for both groups (Fig. 3B; F = 338, *p* < 0.0001), however, overweight and obese subjects had increased mean thickness compared to lean controls (t = 2.2, *p* = 0.03). There was no age:BMI interaction, even after including Cattell scores as an additional regressor.

To investigate the group differences in cortical thickness further, we performed a per-vertex analysis. There were no statistically significant regional changes in thickness between the groups and no age:BMI interactions at a regional level.

### 3.4 Cognitive scores

Cattell scores were available for 463 of the 473 subjects included in the analysis. Scores displayed a significant nonlinear decline with age (F = 79, *p* < 0.001) and were independently predicted by brain size (t = 4.4, *p* < 0.001), however, there were no trait (BMI) or trajectory (age:BMI) effects between lean and overweight or obese individuals (Fig. 3C).

## 4 Discussion

These results indicate that obesity has a modulating impact on age-related brain structural changes. We thus provide direct evidence of a relationship that has been strongly suggested by prior epidemiological and biological work. Strikingly, the overall effects of obesity are redolent of those seen with normal aging. In showing obesity-related alterations in global brain structure, our data support the idea that, like aging, obesity's impact is widespread across the brain. Specifically, our results indicate that increased body mass has a differential effect on brain tissue type, with differences only observed in cerebral white-matter volume and not cortical gray matter. These effects were determined to be maximal in middle-age (approximately 40 years) and equivalent to an increase in white matter–based brain age of 10 years in overweight and obese adults.

Although the exact biological mechanisms are complex (Arnoldussen et al., 2014, Bruce-Keller et al., 2009, Cai, 2013, Chung et al., 2009), 1 suggestion is that proinflammatory cytokines (such as interleukin 6 and tumor necrosis factor-α) and associated hormones such as leptin, produced by adipose tissue, elicit an inflammatory response in microglia which prompts a self-sustaining feedback loop of more cytokines and more inflammation (Arnoldussen et al., 2014, Griffin, 2006, Wilson et al., 2002, Wisse, 2004). These in turn have been linked to white-matter changes (Bolzenius et al., 2013, Kullmann et al., 2015). This biological mechanism suggests that the initial insult of obesity may lead to self-perpetuating damage, which is manifested as structural changes akin to those seen in normal aging. However, it is also observed that obesity itself increases the susceptibility to neurodegeneration (Sriram et al., 2002). Indeed, epidemiological studies suggest that obese people have increased complications and mortality associated with traumatic brain injury compared with lean subjects (Chabok et al., 2013). Thus, it may be that obesity represents an initial insult to the brain that precipitates a cascade of pathologic changes or that it leads to an increased susceptibility to normal aging mechanisms.

Interestingly, our data suggest that middle-age (approximately 40 years) rather than later life may represent a particular period of vulnerability to the effects of increased adiposity. Multiple studies have linked the onset of white-matter changes to middle-age (Bartzokis et al., 2004, Fotenos et al., 2005), and indeed, previous analyses have also related later life structural and cognitive changes to vascular risk factors in midlife (Debette et al., 2011). Moreover, white-matter hyperintensities, a common marker of normal aging, are not usually present in adults before midlife, further emphasizing this as a period of rapid age-related changes (Hopkins et al., 2006). The susceptibility of cerebral white matter to adiposity-related influences may be related to the biology of oligodendrocytes, which continue to differentiate into the 50th decade and are particularly vulnerable to insults (Bartzokis, 2004). The finding that increased body mass equates to an average brain age increase of 10 years further stress the need to tackle obesity, particularly in early adult life. Interventions such as caloric restriction indicate the potential efficacy in preventing or amelioration normal age-related degeneration (Colman et al., 2014). Other studies have suggested that socioeconomic and lifestyle factors which covary with obesity such as income (Sattler et al., 2012), education (Stern et al., 1992), and exercise (Radak et al., 2010, Scarmeas et al., 2009) have all been associated as risk factors for cognitive decline or increased risk of neurodegeneration, however, our analysis failed to find such links. We did find that self-reported hypertension was significantly negatively associated with white-matter volume, suggesting that comorbidities associated with obesity may independently influence neurogeneration. Moreover, although we confirmed that age and brain structure were significant independent predictors of cognition, we did not find a mediating pathologic effect of body mass on this relationship.

Normal age-related white-matter breakdown has been observed independent of changes or loss of neurons or synapses suggesting that white-matter variations associated with obesity do not necessarily imply associated cortical changes (Bartzokis et al., 2004). This is in line with our results which demonstrated a differential effect of adiposity on cortical gray and cerebral white matter. However, unexpectedly, our cortical thickness measures indicated significantly less thinning in overweight or obese subjects compared with lean controls. Why this might be is unclear. One possible explanation is that the accuracy of the thickness measures are compromised by myelination changes associated with normal aging, which affect the gray-white contrast ratio. As explored elsewhere (Grydeland et al., 2013, Storsve et al., 2014, Westlye et al., 2009), this may have the effect of blurring the boundary between gray and white matter, leading to an artifactual increase in measured cortical thickness. If such myelin changes are augmented in obesity, it may be that this will give rise to apparent reduced rates of cortical thinning with age in overweight and obese subjects. In our experiment, we attempted to account for the possible myelin-related confounds on cortical thickness. However, if such effects are significantly increased beyond that observed in normal aging, it may be that this correction is insufficient. In summary, although it is possible that adiposity is associated with an increase in cortical thickness, we must conservatively consider the possibility that these results reflect an artifact of tissue contrast as a product of demyelination effects. The possible confounds associated with cortex-based measures in obesity, as well as the differential effects of its comorbidities on white matter (Verstynen et al., 2013) highlight the subtleties in assessing BMI-related effects on brain structure.

To date there is some evidence in support of the obesity paradox in terms of morbidity and mortality, in that some studies seem to suggest that obesity may in fact be protective. However, the specific relation to neurodegeneration is unclear. Indeed, although some studies have suggested that weight loss may actually precede dementia (Knopman et al., 2007), other studies suggest that increased adiposity is linked to poorer cognition (Whitmer, 2005). In this study, we failed to find any such link using the Cattell battery, which is used to capture fluid intelligence by measuring abstract reasoning ability. Although crystallized intelligence increases with age, fluid intelligence decreases with declining brain function (Horn and Cattell, 1967, Salthouse, 2009). Although previous studies have linked white-matter integrity, processing speed and fluid intelligence (Kievit et al., 2016), our results suggest that BMI does not additionally influence the age and brain structure relationship with cognition. More generally, differences in demographic, clinical (e.g., cognitive status), and socioeconomic variables controlled for may also contribute to the heterogeneity in the literature regarding the relationship between adiposity and neurodegeneration in population-based studies. Similarly the precise way in which adiposity is measured may also be an important consideration. In this study, we used the commonly applied and readily measured variable BMI, however, recent studies indicate that adiposity measured in this way may misclassify subjects as cardiometabiolocally unhealthy (Tomiyama et al., 2016). Moreover, BMI is insensitive to the more clinically relevant distribution of fat on the body, and thus may mask important effects. For example, although waist circumference has been shown to be predictive of cognitive decline, overall obesity has been demonstrated to be neuroprotective in the same sample (West and Haan, 2009). In this study, the absence of more direct measures of relevant health parameters, it is not clear whether our results reflect a relationship between increased adiposity and white-matter volume, or whether BMI is simply a proxy for more fundamental covariates. The use of BMI as a measure of adiposity in this study must be considered a limitation. Finally, the omission of extremely obese subjects (due to scanner limitations) may also be considered to be a limitation of this analysis, potentially obfuscating the true scale of the effect of adiposity on brain age.

Finally, it is important to acknowledge the cross-sectional nature of this analysis and the associated limitations when trying to infer rates of brain aging. Although it is not possible to definitively state that obesity is associated with an increased rate of neurodegeneration, our results however do indicate that across the adult lifespan, an increase in body mass is associated with significantly less cerebral white-matter volume compared with age-matched lean controls and that this change is augmented with increasing age. Previous studies have established the similarity between cross-sectional and longitudinal results when assessing brain structural change with age (Fotenos et al., 2005), which may support the hypothesis that increased adiposity may be associated with increased rates of brain aging, however, a longitudinal analysis taking into account change in body mass as well as brain structure is required to fully establish this link.

## 5 Conclusion

In the global climate of an increasingly aged population, with rising levels of obesity, it is critical to establish the full health impact of an increased body mass. The results of our study suggest that increased adiposity has a significant impact on brain structure that it modulates the relationship between white-matter volume and age and that such effects may be equivalent to an increase in brain age of up to 10 years in overweight and obese individuals. These results support the hypothesis that adiposity confers a significant risk of neurodegeneration and cognitive decline.

## Disclosure statement

Paul C Fletcher has received money in the past for ad hoc consultancy services to GlaxoSmithKline. All other authors have no conflicts of interest to disclose.

## Figures and Tables

**Table 1:** Demographic information

Variables	Lean	Overweight	Obese	*p* for heterogeneity
BMI (kg/m^2^) (mean)	18.5–24.99 22.7 ± 1.7	25–29.99 27.1 ± 1.6	≥30 33.5 ± 3.8	
No. of subjects (%)	246 (51)	150 (31)	77 (18)	
Sociodemographic variables				
Age (years)	48 ± 16	57 ± 17	61 ± 16	<0.0001
Female/male	122/124	66/84	49/28	<0.0001
University degree or higher	180	89	33	<0.0001
Household income (above median)	149	84	38	0.1
Health behaviors				
Current smoking (%)	16	11	6	0.9
Physical activity (kJ/d/Kg)	47 ± 20	47 ± 22	43 ± 23	0.44
Health measures				
Systolic blood pressure (BP) (mm Hg)	116 ± 15	123 ± 16	126 ± 19	<0.0001
Diastolic BP (mm Hg)	71 ± 10	75 ± 11	77 ± 11	<0.0001
Disease diagnosis				
Myocardial infarction	1	3	1	0.3
Cancer	11	6	9	0.03
Diabetes	3	6	9	<0.0001
Stroke	4	0	1	0.3
High cholesterol	21	17	17	<0.01
High BP	19	30	29	<0.001
