# Supplementary material for: Obesity associated with increased brain age from midlife
Source: Neurobiol Aging. 2016 Nov;47:63–70. doi: 10.1016/j.neurobiolaging.2016.07.010 (PMC5082766; doi:10.1016/j.neurobiolaging.2016.07.010)
Supplement: Supplementary Figures 1 and 2 [file mmc1.docx]

***Supplementary Material***

**Figure S1** *Bland and Altman (1986) repeatability plots for (a) white matter volume, (b) cortical surface area and (c) cortical thickness.*

**Figure S2** (a) Spline models of white matter volume revealed a significant single outlier in the overweight / obese group at 20 years (highlighted yellow box). (b) Removal of this outlier generated an inverted U-shaped trajectory for this group. Removal of the outlier did not change statistical results reported.

**
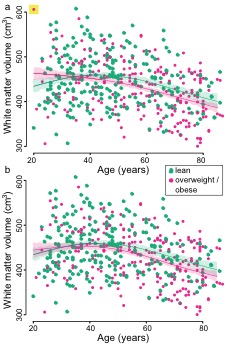
**
